# Accelerated immunosenescence in rheumatoid arthritis: impact on clinical progression

**DOI:** 10.1186/s12979-020-00178-w

**Published:** 2020-03-09

**Authors:** Moisés E. Bauer

**Affiliations:** Laboratory of Immunobiology, School of Health and Life Sciences, Pontifical Catholic University of Rio Grande do Sul (PUCRS), Av. Ipiranga, 6681, Porto Alegre, RS 90619-900 Brazil

**Keywords:** Rheumatoid arthritis, Ageing, Cell senescence, Immune ageing, Cognitive impairment

## Abstract

Patients with rheumatoid arthritis (RA) develop features of accelerated ageing, including immunosenescence. These changes include decreased thymic functionality, expansion of late-differentiated effector T cells, increased telomeric attrition, and excessive production of cytokines (senescence-associated secretory phenotype). The progression of RA has been associated with the early development of age-related co-morbidities, including osteoporosis, cardiovascular complications, and cognitive impairment. Here I review data supporting the hypothesis that immune-senescence contributes to the aggravation of both articular and extra-articular manifestations. Of note, poor cognitive functions in RA were associated with senescent CD28- T cells, inflammaging, and autoantibodies against brain antigens. The pathways of immune-to-brain communication are discussed and provide the rationale for the cognitive impairment reported in RA.

## Introduction

Rheumatoid arthritis (RA) is a systemic chronic inflammatory disease, causing symmetrical and destructive inflammation in joints as well as in multiple tissues. It occurs in approximately 1% of the world’s population and is more frequent in women than in men, at a 3:1 ratio [1]. In the initial phase of the disease, patients develop mostly articular manifestations, particularly in the knee and hand synovial joints. During disease progression, RA is also associated with extra-articular manifestations including, among others, cardiovascular disease, vasculitis, rheumatoid nodules, and cognitive impairment [2, 3].

RA is an autoimmune disease and its pathogenesis includes the recognition of self-antigens that are locally expressed in the synovial tissue. Candidate antigens include type II collagen, the proteoglycans, and the cartilage protein gp39 [4–6]. These antigens have been clearly implicated in autoimmunity and the development of experimental arthritis in rodents. However, their importance for RA pathogenesis has not been unequivocally established in humans. The aetiology of RA has multifactorial contributions of both genetic and environmental triggers to the onset and development of the disease. The presence of certain alleles in the human leukocyte antigen (HLA)-DRB1 gene, such as DRB1*04:01, are strongly associated with RA [7]. These risk alleles encode a five amino acid sequence referred to as a shared epitope at positions 70–74 on the HLA-DR β chain. The presence of the shared epitope in HLA favours the binding of self-proteins that have undergone post-translational modification in a process referred to as citrullination – a physiological process that can be intensified by the presence of a risk allele in the PTPN22 gene, which is also a genetic risk factor for RA, as well as exposure to tobacco smoke [2]. In addition, gender (female), advanced age, and certain foods have been associated with increased risk for developing the disease [8].

Currently, the development of RA is divided into three stages of progression that can be described as follows: (1) preclinical phase, (2) clinical phase and (3) extra-articular manifestations. The first stage comprises the onset of autoimmunity, without clinical manifestations. In the second phase, the dysregulation of regulatory mechanisms, marked by the reactivity of B and T cells against self-antigens, triggers strong inflammatory responses that highlight the clinical onset of the disease. As the disease progresses, joint and extra-articular manifestations become noticeable (3rd stage) [9].

There are two major mechanisms involved in the immunopathogenesis of RA. First, the intimal lining of the joint greatly expands leading to the increase and activation of synoviocytes. These cells secrete several pro-inflammatory cytokines, including tumour necrosis factor (TNF), interleukin (IL)-1, IL-6, proteinases (e.g., metalloproteinases or MMPs) and lipid mediators like prostaglandins and leukotrienes [1]. The synovial invasion into adjacent articular structures damages the cartilage and bone and is a hallmark of RA, characterized by joint swelling. Second, hyperplasia of the synovial layer contributes to the recruitment of neutrophils and lymphocytes (mostly T and B cells). The infiltration of these inflammatory cells has a critical role in RA as they secrete cytokines and proteinases that further degrade the extracellular matrix. Effector CD4+ T cells play a crucial role in the disease, and RA is commonly characterized by an imbalance of Th1/Th17 and regulatory T (Treg) cells [10]. The combined action of these two major mechanisms promotes joint destruction and bone erosion.

## Premature immunosenescence in RA

RA is associated with several features of accelerated aging, including premature immunosenescence and increased prevalence of age-related diseases. The most frequent comorbidities observed in RA patients include cardiovascular disease, malignancies, lung disease, osteoporosis, changes in body composition and neuropsychiatric diseases [11]. Immunosenescence has a special impact on the development of these co-morbidities, as they are all immune-mediated conditions. Nine biological hallmarks have been proposed for aging [12], and can be identified chronologically earlier in subjects with RA: (1) genomic instability (e.g., more DNA damage), (2) telomere shortening, (3) gene regulation (e.g., epigenetic changes), (4) loss of protein homeostasis (e.g., impaired autophagy), (5) altered nutrient sensing (e.g., decreased levels of IGF-1, FOXO, AMPK, mTOR), (6) mitochondrial dysfunction [e.g., mtDNA mutations and reactive oxygen species (ROS) generation], (7) cellular senescence (e.g., altered expression of p16-p53), (8) stem cell exhaustion (e.g., impaired generation of hematopoietic stem cells), and (9) altered intercellular communication (e.g., inflammaging) [13]. This section reviews the changes in both innate and adaptive immune system in RA that resemble those observed in healthy aging. The question whether premature immunosenescence is a primary cause of RA or secondary to the chronic inflammatory processes remains to be answered.

Premature immunosenescence in RA is established by the deterioration of the regenerative capacity of T cells. T cell precursors are produced and undergo differentiation in the thymus. The thymus reaches its maximum functional capacity during puberty, and thereafter undergoes progressive thymic involution associated with the replacement of functional thymic tissue by adipose tissue [14]. This change translates into impaired production and delivery of new naive T cells into the bloodstream. Thymus functional capacity can be estimated by the frequency of peripheral cells expressing T-cell receptor excision circles (TRECs). TRECs are extra-chromosomal DNA sequences that form during T cell receptor rearrangement (TCR) and are diluted by half at each cell division [15]. RA patients show a reduction, regardless of chronological age, in the frequency of TRECS in peripheral blood mononuclear cells (PBMCs) that provides an indirect estimate of the functional integrity of the thymus [16]. Indeed, CD4+ T cells containing TRECs were significantly reduced in RA patients and their TREC levels matched those of healthy controls 20 years older [17]. Moreover, the decreased thymic functionality results in peripheral compensatory mechanisms, such as increased homeostatic proliferation, in order to keep the T-cell compartment quantitatively intact.

RA is also associated with expansion of immune cells with advanced replicative stress. Replicative stress results from the increase in the replicative history of peripheral T cells (homeostatic proliferation / oligoclonal expansion) in order to compensate for the reduction in cell supply due to thymic involution. Aging is associated with a reduction in T-cell receptor diversity, loss of expression of the CD28 costimulatory molecule, and telomeric erosion. CD28 is a cell surface molecule that is required for complete T-cell activation and proliferation. A significant expansion of CD4 + CD28- and CD8 + CD28- in RA was reported many years ago [18, 19], as similarly reported in ageing studies [20]. Of note, patients with extra-articular manifestations of RA had increased frequencies of such cells [21]. The frequency of CD4 + CD28- T cells in these individuals may represent more than 50% of all circulating CD4+ T cells [22]. Anti-TNF therapy was able to significantly reduce CD8 + CD28- T cells (but not CD4 + CD28- T cells) and correlated with clinical response as measured by DAS28/C-reactive protein (CRP) [23]. These are clonal expansions related to significant contractions of the T-cell repertoire. RA patients had ~ 10-fold contraction of their naive CD4+ T-cell repertoire as compared to age-matched controls [24] – indicating that 40–50-year-old RA patients have already lost approximately 90% of their available TCRs. Furthermore, these data indicate that the remaining naïve T cells had to expand to 10 times larger clonal sizes in order to compensate.

Replicative stress of cells may lead to cellular senescence. The cells that have reached replicative senescence do not proliferate but remain metabolically active and acquire new inflammatory and cytotoxic characteristics. The pool of CD28- T cells includes effector-memory and terminally differentiated memory cells re-expressing CD45RA (TEMRA), which may contribute to inflammaging by secreting large amounts of interferon (IFN)-γ, TNF-α, IL-1β, and IL-6 upon stimulation [25]. This mixture of cytokines has been termed the senescence-associated secretory phenotype (SASP). In addition, it has been shown that CD28- T cells acquire NK cell receptors and have high levels of granzymes and perforins, explaining the cytotoxic potential of these cells [26]. It has been proposed that acquisition of a cytotoxic phenotype in senescent T cells may be compensatory for losses observed in T and NK cells [27]. Moreover, a recent study proposed the existence of a subset of senescent Treg cells in the peripheral environment of RA [28]. These cells were defined as CD4 + CD28-Foxp3+ T cells and were positively associated with age and with clinical parameters such as disease activity and treatment. These senescent Treg cells display less suppressive capacity when compared to CD28+ Treg cells [28]. It should be noted that RA is also associated with accumulation of other senescent cells. Indeed, senescent synovial fibroblasts (p16INK4a + cells) accumulate prematurely in RA and display an enhanced inflammatory phenotype [29].

Telomeric erosion has been found to be accelerated in patients with RA, regardless of age. RA patients had increased telomere shortening in granulocytes, PBMCs and CD4+ T cells [17, 30]. During healthy aging, the telomeric length of peripheral lymphocytes shortens 20–40 bp / year. In contrast in RA, this accelerated rate represents 15X the value observed in age-matched healthy donors [31, 32]. Hematopoietic stem cells, granulocytes and lymphocytes are the leukocytes particularly affected in the course of the disease. Overall, the telomeres remain stable until the fourth decade of life in healthy individuals. From 41 to 65 years, a marked reduction reaches the plateau after 65 years of age. In the first forty years of life in RA, the telomeric length is significantly shorter. The faster telomere erosion observed in circulating lymphocytes of RA is due to multidimensional mechanisms including telomere shortening already present in hematopoietic progenitor cells (CD34+), persistent exposure to the inflammatory environment, increased oxidative stress and homeostatic proliferation [32]. However, a recent study reported similar telomere lengths in PBMCs of RA patients (controlled or active disease) and healthy controls [33]. Telomere erosion and reduced telomerase activity in CD4+ T cells were not affected by treatment of RA patients with methotrexate (MTX) or prednisone [17, 34]. PBMCs constitute a mixture of monocytes, B cells, NK cells and T cells, and the telomere erosion may be more evident in isolated CD4+ naïve T cells. It is known that telomere lengths may differ per cell subset and stage of differentiation. For instance, memory B cells have relatively stable telomeres [35]. In addition to increased telomere shortening, CD4+ T cells in RA also have damaged telomeres resulting from defective activity of the DNA break repair nuclease MRE11A [36]. The MER11A^low^ T cells are hypermotile, tissue-invasive, and arthritogenic in vivo (i.e., leading to destructive synovitis).

In gerontological studies, the human cytomegalovirus (CMV) has been shown to accelerate some features of immunosenescence, of note in promoting the expansion of senescent T cells (CD28-), implicated in the reduced T-cell repertoire [37, 38]. Persistent viral infections have long been discussed in the aetiology of several autoimmune diseases, including RA [39]. Previous reports have associated increased CMV seropositivity with expansion of CD4 + CD28- T cells in RA, multiple sclerosis (MS) and systemic lupus erythematosus (SLE) [16, 33]. The CMV-specific CD4 + CD28- T cells were found to be expanded in CMV+ but not in CMV- RA patients [40]. Treatment with ganciclovir in CMV+ RA patients with vasculitis reduced the number of CD4 + CD28- T cells – suggesting that these cells are driven by CMV infection [41]. Methotrexate treatment of RA has also been shown to reduce the levels of CD4 + CD28- T cells [42]. Furthermore, an association with CMV and worsen disease progression and extra-articular manifestations in RA has been reported [43, 44]. It has also been shown that RA patients exhibit CMVpp65-specific IFN-γ production in vitro with expansion of CD28-CD8+ T cells, indicating an efficient control of latent CMV and regardless of current therapy [45]. It has been shown that RA patients had a multi-reactive anti-herpes IgM profile, which was associated with disease activity [46]. Also, expansions of CD28- T cells have also been documented in various chronic infections, including malaria [47], HIV [48] and human T-lymphotropic virus type-I (HTLV-1) [49]. More studies are necessary to explore the role of persistent infections during clinical progression in RA.

Can early immunosenescence be detected in the preclinical phase of RA, or is it a consequence of established disease? It has been reported that healthy individuals genotyped for DRB1*04, a haplotype associated with RA, share with patients the accelerated erosion of telomeres, beginning during the second to third decades of life [30]. These data suggest that chronic inflammation may not be a principal cause of premature ageing in RA. The presence of anti-citrullinated antibodies (ACPA), rheumatoid factor (RF) and high CRP levels in some patients years before onset of clinical symptoms implies that immune responses involved in the development of RA appear very early. However, it is completely unknown whether premature immunosenescence is present in the preclinical phase (ACPA+ arthralgia) of RA. Future prospective studies should be performed to explore this possibility.

## Impact of early immunosenescence on clinical manifestations in RA

**Fig. 1:**
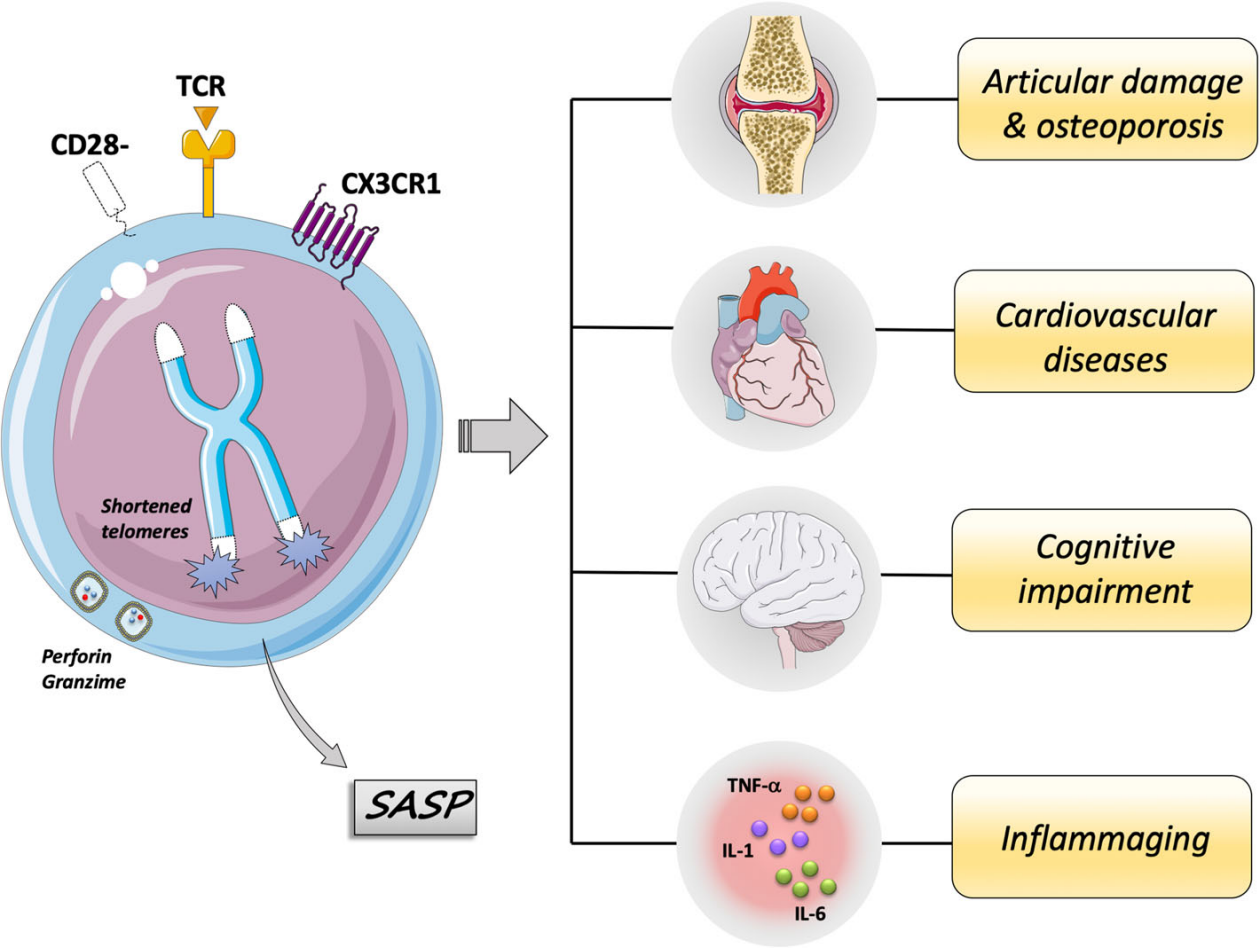
The senescent T cells are associated with inflammaging and age-related morbidities in RA. The late-stage differentiated (senescent) T cells are defined by phenotypic changes including loss of CD28 expression, acquisition of inflammatory (SASP: senescence-associated secretory phenotype) and cytotoxic functions, as well as expression the chemokine receptor CX3CR1, which could underlie their ability to infiltrate peripheral inflammatory sites. These cells do not proliferate, because of shortened telomeres, but remain metabolic active. These cells have been found expanded in RA, of note during the clinical progression. They have been implicated with articular damage and osteoporosis, cardiovascular diseases and cognitive impairment

The immune-senescent profile observed in RA may contribute to the development and aggravation of articular and extra-articular manifestations, which take part in disease progression (Fig. 1). The progression of RA has generally been associated with the early development of characteristics and comorbidities common with older adults. Among these, cognitive impairment, cardiovascular complications and osteoporosis stand out as the main age-related co-morbidities of RA [11].

### Senescent T cells may contribute to disease progression and osteoporosis

The expansion of senescent CD28- T cells has been associated with disease severity in RA [50, 51]. Of note, the peripheral senescent CD4+ T cells were associated with worsening of chronic inflammatory responses due to their close relationship with Th1-type cytokine synthesis [52]. The expansion of CD28- T cell subsets is particularly marked in patients with extra-articular manifestations [21]. However, peripheral CD4 + CD28- T cells and cells with the same phenotype in the joint environment may respond differently under stimulation. While circulating cells in peripheral blood produce more TNF-α, synovial resident cells produce more IL-17, an inflammatory cytokine known to be involved with impaired joint integrity [22]. The presence of senescent cells in the periphery as well as in the rheumatoid synovium may thus contribute to the destructive systemic and pathogenic inflammatory responses. In addition to their inflammatory effects, the CD28- T cells have greater expression of the chemokine receptor CX3CR1, which could underlie their ability to infiltrate peripheral inflammatory sites [53]. The ligand of CX3CR1 is fractalkine (FKN) which is expressed by fibroblast-like synoviocytes. The interaction of senescent CD4 + CD28- T cells with synoviocytes costimulates the production of pro-inflammatory cytokines as well as the release of granules [54]. The expansion of the senescent CD4+ T cell population has been associated with increased bone loss and support of the early development of osteoporosis in RA. Indeed, CD4 + CD28- T lymphocytes from RA patients exhibit higher concentrations of the receptor activator of nuclear factor kappa-Β ligand (RANKL) than CD28+ cells [55]. RANKL, in combination with TNF-α, IL-6 and IL-1β, acts on the differentiation of osteoclasts and degrades mineralized bone matrix through protease synthesis [56]. Therefore, the expansion of peripheral senescent CD4+ T cells may impact bone mineral density and are negatively related to osteoporosis [55]. As previously discussed, CMV infection may worsen the clinical course and facilitate the development of extra-articular manifestations via the expansion of CD4 + CD28- T cells [39]. To date no study has investigated the impact of CMV serology on the development of osteoporosis in RA. In addition, the ACPAs and RF are associated with low bone mineral density and osteoclast-mediated bone resorption, independently of inflammation in patients with RA [57, 58]. Of note, bone loss was identified before the clinical onset of RA in subjects with ACPAs [59].

RA patients have reduced bone mineral density as compared to age-matched controls [60]. Bone loss in RA occurs in the early stages of the disease and is intensified with increasing clinical severity. During the first two years from diagnosis, osteoporosis or osteopenia is present in 11 and 24.7% of individuals with rheumatoid arthritis, respectively [61]. Many factors inherent to the immunopathology and therapy of RA are candidate risk factors for the development of osteoporosis. Among these, the use of glucocorticoids, reduced physical activity, high disease activity and high titres of autoantibodies (RF and ACPAs) stand out in the pathological scenario of osteoporosis in RA [60, 61]. These risk factors affect the balance between bone matrix formation and degradation, promoting resorption rates, which in turn result in osteoporosis.

### Cardiovascular events linked with inflammaging and senescent T cells

Mounting evidence indicates that chronic inflammation is a major risk factor for development of cardiovascular complications in the elderly as well as in adults with RA [62–64]. RA patients are at high risk of developing early cardiovascular complications [50, 65]. The risk rate for coronary artery disease and myocardial infarction in patients with RA is 2–3 times higher and can be identified even in the early stages of the disease. These risk rates are similar to those presented by subjects without RA but 10 years older [11, 62]. The increased risk of cardiovascular morbidity in RA can be explained by (1) the chronic systemic inflammation, (2) the modulating effect that RA has on traditional cardiovascular risk factors (e.g., hypertension, smoking, dyslipidemia and obesity), and (3) the use of specific medications, including non-steroidal anti-inflammatory drugs, glucocorticoids, and disease modifying anti-rheumatic drugs [11]. Longitudinal studies have shown a tendency to reduce cardiovascular risk with improved disease activity [65].

The healthy heart contains several leukocytes, including macrophages, neutrophils, B cells, and T cells. The role of cardioimmunology in cardiac homeostasis and disease can be found in a recent comprehensive review [66]. Perturbations in cellular composition or functionality changes in the heart as well as in the atherosclerotic plaque may thus contribute do the onset and progression of cardiovascular diseases. Several studies have linked cardiovascular disease with increasing senescent CD28- T cells. Of note, the CD4 + CD28- T cells have been found expanded in subjects with angina, acute coronary syndrome, myocardial infarction, chronic heart failure and abdominal aortic aneurysms [67–72].

CMV and CMV-specific T cells are also involved in cardiovascular disease [44]. CMV has been associated with cardiovascular disease since it was isolated from atherosclerotic lesions, although it was largely unknown whether it played a causative role [73]. It has also been shown that mice infected with murine CMV (MCMV) developed hypertension within weeks, independently of atherosclerotic plaque formation, resulting from persistent CMV infection of vascular endothelial cells (EC) [74]. CMV leads to the development of CD4 + CD28- T cells with cytotoxic features targeting the vascular endothelium [75]. Because inflammaging plays an important role in the pathology of cardiovascular disease, CMV may also play a direct role in skewing inflammatory responses. There is some evidence supporting this premise. It has been shown that CMV may induce the production of prostaglandin E2 (PGE2) in human fibroblasts and the CMV envelope glycoprotein (gB) upregulates the expression of NF-κB, a key transcription factor for genes encoding inflammatory mediators [76]. Therefore, CMV has both direct and indirect roles (i.e., through the expansion of cytotoxic CD28- T cells) in promoting cardiovascular disease.

Telomere shortening is also associated with co-morbidities related to extra-articular manifestations. Increased telomere attrition was shown in T cells from patients with atherosclerosis and senescent T cells were also reported to be important in hypertension [77]. Telomeric erosion is related to the development of atherosclerotic plaque and cardiovascular disease in individuals with RA [78]. Also, the shortening of the terminal portions of chromosomes is associated with increased reactivity to self-antigens and loss of immunological tolerance, thus it has been suggested as a possible risk factor for the development of RA [79]. However, it remains to be established how senescent T cells are regulated and contribute to the pathogenesis of cardiovascular diseases. It is speculated that increased numbers of senescent T cells with cytotoxic and pro-inflammatory features may directly contribute to cardiovascular disease.

### Cognitive impairment, depression and anxiety

Patients with RA, in active disease or in remission, have significant impairment of cognitive functions in several cognitive dimensions as compared to controls. These include deficits in attention and working memory, processing speed and executive functions, inhibitory functions, and verbal declarative memory [19, 80]. The impairment in cognitive functions is profound and performance of RA patients is reduced to 50% of that observed in age-matched healthy controls. The cognitive impairment was found worsen in patients with active disease as compared with those in remission [80]. Interestingly, cognitive impairment is related to changes in plasma neurotrophins, which are closely involved in cognition and neuroplasticity. Cognitive impairment has also been correlated with more functional limitations, depression and pain in RA patients. Potential risk factors for cognitive impairment are educational level, low income, oral glucocorticoid use and presence of cardiovascular disease [11]. However, previous studies found no associations between cognitive impairment and glucocorticoid use in RA [80–82].

Several mechanisms are potentially involved with cognitive dysfunction in RA. One mechanism is persistent inflammation, which is known to contribute to cognitive decline during healthy aging [83–85]. Another potential mechanism involves the expansion of senescent immune (and non-immune) cells in RA. In particular, it has been shown that the increasing population of senescent CD8 + CD28- T cells was negatively correlated with the memory functions of RA patients [19]. In contrast, RA patients with higher amounts of memory T cells (CD45RO+) had better cognitive function. This finding is in agreement with the fact that cerebrospinal fluid of healthy adults contains large amounts of memory CD45RO+ T cells [86]. CMV infection may interact (indirectly) with cognitive functions in RA through the expansion of CD28- T cells, albeit no study has investigated this interaction. There is a growing interest in the role of infectious agents in the pathogenesis of dementia, but evidence is scarce. A recent meta-analysis explored the effect of any of eight human herpesviruses on development of dementia or mild cognitive impairment (MCI) [87]. Recent infection or reactivation of herpes simplex virus type 1 or type 1/2 unspecified, CMV and human herpes virus-6 was associated with dementia or MCI, though results were inconsistent across studies.

Circulating autoantibodies may also contribute to poor cognition in RA. It has been shown that RA patients in remission had increased levels of autoantibodies targeting brain antigens, such as myelin basic protein and myelin oligodendrocyte glycoprotein, which inversely correlated with cognitive tests [88]. The same study reported increased plasma levels of central nervous system (CNS)-restricted proteins (S100β) in RA, indicating a dysfunctional blood brain barrier (BBB). CNS antibodies are normally restricted to the brain parenchyma. However, increased BBB permeability is frequently observed in chronic inflammatory disorders (including RA) and may thus facilitate the influx of circulating molecules [89]. Increased plasma levels of CNS autoantibodies have also been found in Systemic Lupus Erythematosus and Sjogren’s syndrome, and also associated with poor cognition. The cognitive impairment in RA could be thus explained by the detrimental action of CNS autoantibodies, as suggested by demyelination observed in some RA patients [90]. However, further studies are necessary to investigate to what extent these autoantibodies are related to premature senescence in RA.

Depression and anxiety are also common in RA and associated with worsening disease progression [91]. Also, the presence of depression can aggravate responses to disease-modifying interventions including biological therapies [92]. Mood swings, irritability and panic attacks are frequently reported in autoimmune disorders. The prevalence of depression is considerably higher in RA. A meta-analysis that included 13,189 patients from 72 studies revealed that the prevalence of major depression is nearly 3X higher than expected in the general population [93]. Several clinical trials using monoclonal antibodies (TNF or IL-6 inhibitors) to treat RA resulted in significant antidepressant effects [94]. Indeed, persistent inflammation could be one of the potential mechanisms involved with depression in RA, as solid evidence indicates that pro-inflammatory mediators change key brain areas involved with cognition and mood [95]. Also, it is tempting to speculate that peripheral activated CD4+ T cells may promote anxiety behaviour in RA. As discussed below, stress-induced xanthine by peripheral CD4+ T cells is capable to induce anxiety-like behaviour in mice [96]. Patients with RA have increased serum xanthine levels [96] – and those with the highest levels had good responses to anti-TNF-α therapy [97]. Given that TNF is produced at high levels by RA T cells [98], this pathway is possibly involved in the immune-to-brain communication and in the generation of anxiety behaviour. It remains to be established whether senescent immune cells are contributing to depression in RA. One study reported an accumulation of peripheral senescent T cells (CD57 + CD28-) in older hip fracture patients who developed depressive symptoms [99]. These data indicate that onset of depression after a hip fracture in older adults is an important driver of immune dysregulation. It will be discussed in the next section how plasma inflammatory mediators and peripheral immune cells influence complex human behaviors, impairing cognitive functions and leading to depression.

## The pathways of immune-to-brain communication

**Fig. 2:**
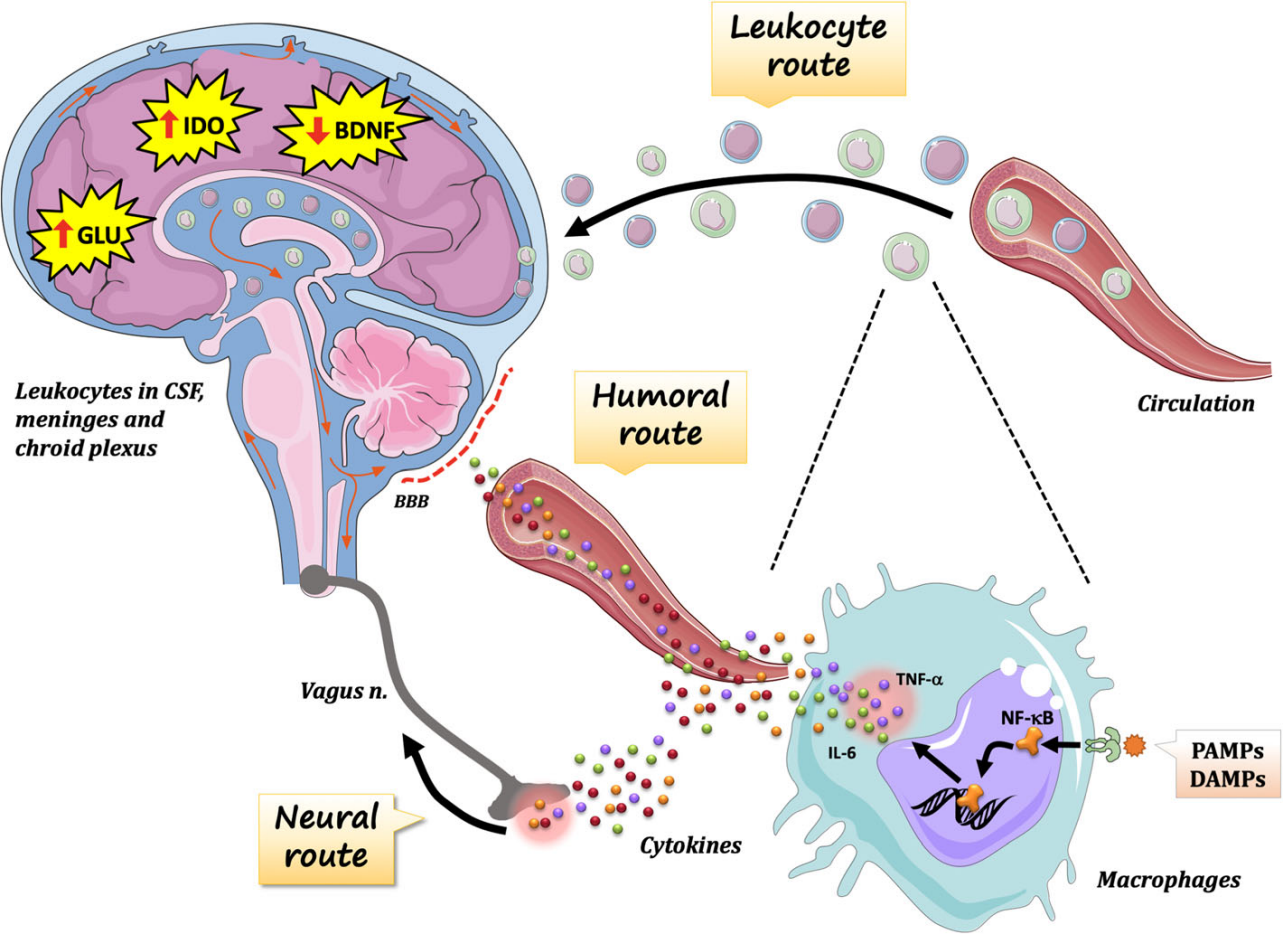
Pathways involved in the immune-to-brain communication. Three pathways participate in the immune-to-brain communication: humoral, neural and cellular (leukocyte) routes. Tissue-resident macrophages get activated by pathogen-associated molecular patterns (PAMPs) or damage-associated molecular patterns (DAMPs). Both PAMPs (infections) and DAMPs (sterile injury) engage inflammatory signalling pathways, such as nuclear factor-κB (NF-κB). The pro-inflammatory cytokines in turn are promptly secreted and enter the bloodstream. The plasma cytokines can reach the brain through various mechanisms, including the **i)** active transport by crossing the brain-blood barrier (BBB) through leaky areas in the circumventricular organs (humoral route); or **ii)** through the activation of afferent neural pathways (e.g., the vagus nerve). The leukocytic route is another mechanism of immune-to-brain communication and is mediated by the migration of circulating leukocytes to the brain borders. Leukocytes are present in small numbers in brain circumventricular organs and choroid plexus. Under healthy conditions, these peripheral immune cells support neuronal function and scan the brain for pathogens or tissue damage

Peripheral immune cells and derived molecules cross-talk to the brain during physiological and pathological processes, modulating key brain processes involved with cognition, mood and social behaviour. This cross talk is established by three pathways: the humoral, neural and cellular (leukocyte) routes (Fig. 2). In the humoral route, inflammatory mediators (i.e., cytokines) reach the brain directly or indirectly through the bloodstream. In the neural route, inflammatory molecules and damage-associated molecules, which are in close contact with peripheral innervation in inflamed tissues, lead to brain changes via activation of afferent neuronal pathways. Finally, circulating leukocytes (mostly T cells and monocytes) secrete cytokines into the cerebral tissue at the brain borders and therefore also interfere with brain processes.

### The humoral pathway of immune-to-brain communication

The “sickness behaviour” is the prototype of humoral pathways for neuro-immune communication. Following an infection, the organism rapidly starts a set of changes to enhance survival. This set of changes may include sickness behaviour, as shown by physiological (e.g., fever, blood composition changes), behavioural (e.g., decreased locomotion, food and water intake) and hormonal changes (e.g., secretion of hormones from the hypothalamic-pituitary-adrenal, HPA, axis). This “sickness behaviour” is then a proxy for a depressive state observed in an acutely ill animal or person. For instance, the set of behaviours observed during the febrile state include depressed mood, impaired cognition, apathy, and irritability – all of which are core symptoms of major depressive disorder. The expression “sickness behaviour” is also manifested in the context of an existing chronic low-grade inflammation as observed in different neuropsychiatric disorders, including major depression, bipolar disorder, and schizophrenia [100]. Sickness behaviour is thus critically related to peripheral pro-inflammatory cytokines.

The increasing levels of plasma pro-inflammatory cytokines observed in RA may directly trigger changes in CNS functions. Plasma cytokines may gain access to the brain via saturable transporters at the blood-brain barrier (BBB) [101]. The “sickness behaviour” can be induced by administration of pro-inflammatory cytokines, or endotoxin. IL-1β and TNF-α are the main cytokines involved in triggering it. In rodents, both systemic and intracerebral administration of IL-1β or TNF-α results in signs of sickness behaviour in a time- and dose-dependent manner. Furthermore, IL-6-deficient mice have attenuated signs of LPS-induced sickness behaviour as compared with wild-type animals, indicating that IL-6 expression in the brain may contribute to the expression of other cytokines (including IL-1β and TNF-α) following immune challenges [101].

How do pro-inflammatory cytokines promote depressive behaviour? The underlying mechanisms involve changes in brain monoamine levels (i.e., dopamine and serotonin), excitotoxicity (i.e.*,* increased glutamate levels) and reduced neuroplasticity. Pro-inflammatory cytokines may reduce brain levels of serotonin, an important neurochemistry alteration in depression and target of some antidepressants. Both experimental studies and clinical trials have indicated that the decrease serotonin levels was highly correlated with the development of cytokine-induced depressive symptoms. Previous studies investigating the effects of IFN-α upon indolamine 2,3 dioxygenase (IDO) pathway provided further evidence that the serotonin pathway is influenced by pro-inflammatory cytokines. IDO is involved in breaking down tryptophan into kynurenine. It is expressed in the brain, and is highly inducible by pro-inflammatory cytokines. Under inflammatory conditions, tryptophan availability for serotonin synthesis decreases, while kynurenine levels increase due to an enhanced IDO activity [101]. Moreover, kynurenine easily crosses the blood brain barrier (BBB) and enters the brain, where it is metabolized by glial cells into 3-hydroxykynurenine (3-HK), quinolinic acid (QA), kynurenic acid (KA). 3-Hydroxykynurenine is an oxidative stressor, whereas QA is an N-methyl-D-aspartate (NMDA) receptor agonist, stimulating glutamate release and blocking glutamate reuptake by astrocytes [102]. Quinolinic acid is also associated with lipid peroxidation and oxidative stress. Taken together, these activities may lead to excitotoxicity and neurodegeneration (key features of mood disorders). In contrast to QA, KA can reduce glutamate and dopamine release, which in turn can contribute to cognitive dysfunction [102]. Increased levels of QA have been found in the brain of suicide victims with depression [103]. Moreover, the binding of glutamate to extrasynaptic NMDA receptors may lead to decreased levels of brain-derived neurotrophic factor (BDNF), impairing neuroplasticity (essential to cognition) [104]. BDNF is crucially involved in neurogenesis, essential for an antidepressant response, and has been shown to be reduced by TNF-α and IL-1β [105]. It has been suggested that neuroinflammation, as observed during ageing or chronic inflammatory diseases, may degrade the BDNF levels needed to maintain cognitive-related plasticity processes at hippocampal synapses [106]. Indeed, previous preclinical studies reported detrimental effects of inflammation on BDNF expression in the brain [107].

Is there any evidence that neurotrophins are altered in RA? In a recent study, RA patients (of note those with active disease) had increased plasma levels of BDNF [80]. Although this finding seems in contrast to what is expected for patients with chronic inflammation, it should be noted that most of the circulating BDNF is likely derived from a leukocyte source in inflammatory disorders. Indeed, previous studies reported increased BDNF levels in RA and Lupus [108]. In addition, it has been demonstrated that PBMCs and synovial cells constitutively express BDNF [109]. However, poor cognition in RA patients was associated with lower plasma levels of glial cell line-derived neurotrophic factor (GDNF). Because GNDF is only produced in the CNS [110], lower levels of this neurotrophin may better predict poor memory performance than BDNF.

### The neural pathway of immune-to-brain communication

Peripheral sensory neurons are found in close proximity to immune cells, and are capable of carrying afferent immune-related signals to the brain via the spinal cord (sympathetic) and vagus nerve (parasympathetic) [105]. The afferent pathway of this communication includes vagal stimulation by inflammatory cytokines, enabling an unconscious representation in the CNS of peripheral inflammation [111]. In this context, the immune system would be acting as a “sensory organ”, a concept originally proposed by J. Edwin Blalock during the 1980’s [112]. The afferent vagus nerve ends primarily in the brainstem medulla. It is then communicated to other brainstem nuclei and forebrain regions associated with integration of visceral sensory information as well as coordinating of autonomic functions and behavioural responses [113].

Moreover, the efferent vagus nerve is known to modulate the immune system - of note inflammation. Although the efferent arc of the vagus nerve does not directly communicate with lymphoid organs [105], its cholinergic stimulation via acetylcholine (ACh) secretion suppresses excessive inflammation in the heart, liver, pancreas and gastrointestinal tract [113]. The efferent arc of the vagal-immune communication is part of the “inflammatory reflex” [114]. It has been shown that signals from the efferent vagus reach the splenic nerve, which induce the release of ACh by a splenic T-cell subset with important anti-inflammatory actions [115]. Interestingly, in nude mice (i.e., lacking T cells) vagal stimulation cannot restrain the inflammatory response. However, the transfer of ACh-producing T cells, repopulating the spleen in nude mice, restores the integrity of this anti-inflammatory neural circuit [115].

Preclinical studies have been performed to explore the efferent arm of the inflammatory reflex in controlling acute and chronic inflammation. The efferent pathway of the inflammatory reflex is responsible for attenuating TNF-α levels during septic shock. Accordingly, a range of sickness responses was abolished by cutting the vagus nerve, including fever, decreased food-motivated behaviour, increased sleep, decreased activity, decreased social interaction, changes in brain activity, and release of stress hormones [116]. ACh interacts with nicotinic receptors (α7), expressed by several types of leukocytes, and the intracellular pathways of this regulation have already been elucidated [117]. Clinical studies have also indicated that innate immune responses (inflammation) are under vagal control. Previous studies with healthy adults revealed that systemic low-grade inflammation (i.e., increased plasma CRP and IL-6) was associated with reduced vagus nerve activity [118, 119]. There is also increasing evidence that vagal control is impaired during chronic inflammatory conditions. Indeed, the association of reduced vagal activity and increased inflammatory responses has been demonstrated in a range of chronic inflammatory diseases, including arthritis, lupus and inflammatory bowel disease [120–122]. Based on these data, clinical trials with electrical stimulation of the vagus nerve, through pacemaker-like devices, have yielded positive results in controlling chronic inflammation. Indeed, electrical vagal stimulation (up to four times daily) inhibited TNF-α production for up to 84 days and attenuated disease severity in RA [123].

### The cellular pathway of immune-to-brain communication

Circulating leukocytes, including senescent cells, may also be contributing to cognitive impairment, depression and anxiety reported in RA. The cellular (leukocyte) pathway is a known axis of immune-to-brain communication and includes leukocytes present in small numbers in brain vasculature, choroid plexus and meninges. Under healthy conditions, these immune cells support neuronal functions and may also scan the brain for pathogens or tissue damage [124]. It should be noted that under healthy conditions leukocytes are not found in the cerebral parenchyma. The recruitment of blood monocytes to the brain has been widely reported in classical inflammatory conditions, such as neurotrauma and neuroinfection. This monocyte trafficking into the brain is required for inducing anxiety-like behaviour after neurophatic pain [125], and cognitive impairment following peripheral surgery [126]. Mnocyte migration to the brain was also observed following psychosocial stress [127], and associated with prolonged anxiety behaviour after stress. The accelerated senescence observed in RA could also be involved in brain changes associated with neuroinflammation. Immunosenescence and inflammaging may induce neuroinflammation by modulating glial cells (e.g., microglia) towards a more pro-inflammatory state (M1-like), leading to dysfunctional neural changes and accumulation of brain tissue damage [106, 128].

Peripheral lymphocytes have key roles in regulating healthy brain functions, including responses to psychosocial stress [129], spatial learning and memory [130] and adult neurogenesis in rodents [131]. Of note, autoreactive CD4+ T cells have been implicated in the regulation of cognitive processes and mice deficient in T cells are cognitively impaired [131, 132]. The pro-cognitive properties of T cells have been associated with increased production of IL-4, IFN-γ and IL-17 at brain borders [133–135]. These cytokines were shown to improve learning behaviour via the upregulation of BDNF expression by neural cells. In addition, it has been shown that CD4+ T cells with defective mitochondria lead to stress-induced anxiety behaviour in mice through the release of xanthine [96]. Patients with anxiety had higher serum xanthine levels than healthy controls. Also, it was shown that T cell-derived xanthine has specific action on oligodendrocytes in the left amygdala, a key brain area involved with stress and anxiety. Interestingly, the mitochondrial pattern observed in these stress-exposed CD4+ T cells is similarly observed in RA CD4+ T cells [136]. These data highlight the importance of secreted products of peripheral CD4+ T cells for the maintenance of cognitive and stress-related functions. In addition, it has been shown that CD4+ T cells expressing the T-box transcription factor Eomesodermin (EOMES) have cytotoxic features and are essential for chronic neuroinflammation observed in experimental autoimmune encephalomyelitis (EAE) [137]. These EOMES+CD4+ T cells were found to be increased in the peripheral blood and cerebrospinal fluid from patients with multiple sclerosis. These cells may be also relevant for the pathogenesis of RA, as EOMES+CD4+ T cells were found expanded in the synovial fluid of RA patients [138]. It remains to be investigated whether these cells modulate cognitive functions in RA.

Senescent CD28- T cells may be also implicated in modulating cognitive processes, as expansion of this subset has been associated with cognitive impairment in RA [19, 80]. The expansion of CD28- T cells has been also reported in neuropsychiatric disorders associated with cognitive impairment, including bipolar disorder, early-life stress, and Alzheimer’s disease [139, 140]. Indeed, patients with mild cognitive impairment or Alzheimer’s disease had increased expansion of late-differentiated T cells (CD28-) [141, 142]. These data further support the role of peripheral lymphocytes in human cognition. Although the underlying mechanisms by which senescent T cells modulate brain processes were not elucidated, it is tempting to speculate it could be related to their pro-inflammatory phenotype.

## Conclusions

In this review, I have attempted to give a perspective on key aspects of accelerated ageing in RA. The hallmarks of ageing can be identified prematurely in RA and clinical progression is associated with development of age-related co-morbidities. Patients with RA also show several signatures of accelerated immune ageing, of note in ageing T cells. Briefly, immunosenescence is characterized by the profound thymic involution, enhanced telomere erosion, defective telomerase activity, increased CMV seropositivity, contraction of the T-cell repertoire and accumulation of late-stage differentiated T cells (CD28-). The question whether premature immunosenescence is a primary cause of RA or secondary to chronic inflammatory processes remains to be answered. More prospective studies should be performed to study immunosenescence in preclinical RA and to explore the association with RA severity and development of extra-articular manifestations.

Osteoporosis, cardiovascular complications and cognitive impairment stand out as the main age-related co-morbidities of RA. Immune cells participate actively in the healthy physiology of articular, cardiovascular and brain tissues. Changes in the immune cells taking part in the peripheral tissues (including the brain) have thus been implicated in pathological processes. Indeed, senescent T cells have been associated with disease progression and development of age-related diseases in RA as they have pro-inflammatory (i.e., SASP) and cytotoxic phenotypes as well as displaying enhanced ability to infiltrate into inflammatory tissues. Senescent T cells, chronic inflammation and autoantibodies targeting CNS antigens have been associated with poor cognition in RA. There is some evidence that CMV may be involved in the clinical manifestations in RA, of note in cardiovascular disease. Finally, this article reviews data supporting the hypothesis that peripheral immune cells and derived molecules cross-talk to the brain, and modulate key processes involved with cognition, mood and social behaviour. This cross talk is established by three pathways: the humoral, neural and leukocyte routes. Hence, the removal of senescent immune cells by new therapies should improve clinical progression in RA.

## Data Availability

Not applicable.

## Abbreviations

ACh: Acetylcholine
ACPAs: Anti-citrullinated protein antibodies
BBB: Blood brain barrier
BDNF: Brain-derived neurotrophic factor
CMV: Cytomegalovirus
CNS: Central nervous system
CRP: C-reactive protein
DAMPs: Damage-associated molecular patterns
FKN: Fractalkine
GDNF: Glial cell line-derived neurotrophic factor
HLA: Human leukocyte antigen
IL: Interleukin
MMPs: Metaloproteinases
MTX: Methotrexate
NK: Natural killer
PBMCs: Peripheral blood mononuclear cells
RA: Rheumatoid arthritis
RANKL: Receptor activator of nuclear factor kappa-Β ligand
RF: Rheumatoid factor
SASP: Senescence-associated secretory phenotype
TEMRA: Terminally differentiated memory cells re-expressing CD45RA
TNF: Tumour necrosis factor
TRECs: T-cell receptor excision circles
Treg: Regulatory T
